# The Developmental Dynamics of Terrorist Organizations

**DOI:** 10.1371/journal.pone.0048633

**Published:** 2012-11-21

**Authors:** Aaron Clauset, Kristian Skrede Gleditsch

**Affiliations:** 1 Department of Computer Science, University of Colorado, Boulder, Colorado, United States of America; 2 BioFrontiers Institute, University of Colorado, Boulder, Colorado, United States of America; 3 Santa Fe Institute, Santa Fe, New Mexico, United States of America; 4 Department of Government, University of Essex, Wivenhoe Park, Colchester, United Kingdom; 5 Centre for the Study of Civil War, Oslo, Norway; Umeå University, Sweden

## Abstract

We identify robust statistical patterns in the frequency and severity of violent attacks by terrorist organizations as they grow and age. Using group-level static and dynamic analyses of terrorist events worldwide from 1968–2008 and a simulation model of organizational dynamics, we show that the production of violent events tends to accelerate with increasing size and experience. This coupling of frequency, experience and size arises from a fundamental positive feedback loop in which attacks lead to growth which leads to increased production of new attacks. In contrast, event severity is independent of both size and experience. Thus larger, more experienced organizations are more deadly because they attack more frequently, not because their attacks are more deadly, and large events are equally likely to come from large and small organizations. These results hold across political ideologies and time, suggesting that the frequency and severity of terrorism may be constrained by fundamental processes.

## Introduction

Much research on patterns in terrorism has been inspired by particular historic events and “waves” of specific forms of terrorist attacks [1], [2]. Just as the rise in international skyjackings in the 1970s led to a resurgence of studies of terrorism, the 11 September 2001 attacks renewed interest in why groups resort to terrorism, the specific choice of attack targets, and the relative effectiveness of particular counterterrorism measures. As a result, many researchers have developed typologies of specific forms of terrorism and highlighted the distinctiveness of different terrorist groups. By contrast, in this manuscript we examine whether there are fundamental patterns in the frequency and severity (number of deaths) of deadly events carried out by terrorist organizations and what mechanisms might generate them.

Little research on terrorism has focused on directly modeling individual event frequency and severity, and the way these change over an organization's lifetime. When deaths are considered, they are typically aggregated and used as a covariate to understand other aspects of terrorism, e.g., trends over time [3], [4], the when, where, what, how and why of the resort to terrorism [5]–[7], differences between organizations [8], or the incident rates or outcomes of events [3], [9]. Such efforts have used time series analysis [3], [4], [9], qualitative models or human expertise of specific scenarios, actors, targets or attacks [10] or quantitative models based on factor analysis [11], [12], social networks [13], [14] or formal adversarial interactions [6], [15], [16].

Our approach is different and complementary to these approaches, focusing on global trends and patterns in the frequency and severity of events [17]–[25], rather than on event particulars or motivations. By focusing our analysis at the global scale, the importance of individual decisions in specific contexts is in fact lessened, due to the central limit theorem and the rough independence of individual events; as a result, the importance of generic non-strategic processes is enhanced and these processes, if any, may be studied. Explanations of such patterns must thus focus on processes or constraints that are independent of variations in context or specific motivation and may include physical constraints, network effects and endogenous population dynamics, which are well suited to explain the behavior of strategically uncöordinated populations of actors [24]. This approach to investigating the fundamental laws of terrorism has much in common with that of statistical physics, in which the self-averaging properties of independent events allows for interesting population-level properties to emerge from microscopic system chaos. This statistical physics-style approach is increasingly being applied to study complex social systems [26]–[28], yielding a number of novel insights.

Here, we aim to shed new light on the fundamental processes governing the frequency and severity of terrorist events by studying their statistical relationship with the organizations that generate them. Our aim is to identify global patterns in these relationships and to explain their origin mechanistically. We employ a combination of disaggregated data analysis, studying a large database of terrorist events worldwide from 1968–2007, statistical modeling and inference, computational modeling and regression analysis to validate our mechanistic hypotheses. By shedding new light on these large-scale patterns and trends in terrorism, and on how such patterns emerge from local-level behaviors, this large-scale statistical or pattern-based approach can supplement formal models of strategic interactions, inform counter-terrorism policy and clarify our general ability to forecast or anticipate future terrorist events or trends.

### Patterns in global conflict

A pattern-based approach to studying conflict owes much to the seminal work in the early 20th century of Lewis Fry Richardson–a physicist and meteorologist known for collecting data on conflicts (“deadly quarrels”), modeling arms races using differential equations, as well as early contributions to understanding the frequencies and severities of wars. Specifically, Richardson [29], [30] identified the remarkable pattern that the frequency of wars decays like the inverse power of their severity. (Power-law distributions can indicate unusual underlying or endogenous processes, e.g., feedback loops, network effects, self-organization or optimization. From a purely statistical perspective, power-law distributions generate large events orders of magnitude more often than we would expect under a Normal assumption. Recently, power-law distributions have been identified in a wide range of social and biological systems [31]. See [32], [33] and [34] for reviews, or Appendix A of [35] for a gentle introduction.) This empirical pattern implies that there is no fundamental statistical difference between rare but catastrophic wars and more common but less severe wars–the likelihoods of both are described by a single mathematical function:
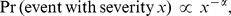
where 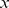 counts the number of fatalities (severity) and 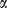 is the “scaling exponent,” which controls how quickly the frequency decreases as severity increases. It also implies that the underlying social and political processes for both large and small wars may be fundamentally the same, i.e., large wars may simply be “scaled up” versions of small wars. In general, the identification of a power law implies that studying the statistically more common events can shed light on certain aspects of extremely rare events. (Seismologists study large earthquakes in this way: the frequencies of both large and small quakes follow a power-law distribution, called the Gutenberg-Richter Law, and the physical processes that generate both small and large quakes are fundamentally the same).

Recently, Clauset et al. [20], [31] showed that this same pattern–a power-law, “Richardson's Law”–also holds for the frequency of severe terrorist attacks (reported fatalities) worldwide, while [23] suggest a similar pattern for events within insurgencies. The power-law pattern in terrorism is highly robust: it persists over the past 40 years despite large structural and political changes in the international system and is independent of the type of weapon used (explosives, firearms, arson, knives, etc.), the emergence and increasing popularity of suicide attacks, the demise of many individual terrorist organizations, and the economic development of the target country.

Thus, fundamental regularities in terrorism can and do emerge at the global level despite the highly contingent and context-specific nature of the individual attacks, conflicts and decisions. Insights into how these patterns' arise will likely shed new light on the underlying social or political processes that drive and constrain global trends and on effective policies for responding to or managing those processes.

## Methods

We consider the frequency and severity of attacks over the lifetime of individual terrorist organizations, and the question of whether organizations exhibit common statistical patterns in these behaviors. We argue that organization size (number of personnel) plays a fundamental role in limiting the overall frequency, but not the severity, of violent events by a group. The key idea is that organization size and its overall production rate of events are linked. If events lead to growth in any way, then this link implies a positive feedback loop in which each attack increases the production rate of future attacks. Thus, a terrorist organization can be viewed as a kind of factory whose principal product is political violence, and whose proceeds are reinvested in increased production capacity.

To test these “developmental dynamics” hypotheses, we present novel statistical analyses of the behavior of nearly 400 terrorist organizations worldwide over the period 1968–2008. We find strong evidence for precisely this kind of generic acceleration in event production. This supports the notion that an organization's available labor, i.e., the size of its militant wing, is a fundamental constraint on the overall frequency of its attacks. We further show that the rate at which an organization cycles through the positive feedback loop can depend on covariates like its political ideology, with religiously-motivated organizations accelerating (growing) the fastest. In contrast, we find no evidence that event severity depends on organizational size or experience. Instead, the distribution of attack severities follows a rough form of Richardson's Law independent of size, experience or political motivation.

These results imply that very large events are equally likely to be generated by small groups as by large groups, and that larger organizations are indeed more deadly [8], not because their individual attacks are systematically more spectacular but because they typically carry out many more attacks. That is, the size of the beast directly determines the overall level of terror activity (frequency) but not the quality (severity) of those actions.

Recently, Johnson et al. [25] used a similar approach to analyze the timing of events in the Iraq and Afghanistan conflicts, which was in turn based on an earlier version of this manuscript [22]. Although similar statistical patterns to the ones we describe here were observed in those conflicts, a different explanation was offered for their origin. We will revisit this comparison and comment on the problems our statistical results pose for the explanation offered by [25].

### Impact of Size on Frequency

H1 *Labor-constraints*: the overall production rate of violent events by an organization depends on its size, and thus the time between consecutive attacks Δ*t* is roughly inversely proportional to the size *s* of the organization. Mathematically, *s∝1/*Δ*t.*

In other words, the production of terrorist events cannot be automated. If this were possible, organizations could produce arbitrary numbers of events without needing to grow in size, much like a fully automated factory requires essentially no human personnel to function. (In this light, *cyber terrorism* is an interesting case: it remains unclear to what degree the planning and execution of cyber terrorist attacks can be done automatically, by computers. Our current belief is that cyber terrorism is also not mass produceable and thus some labor constraint will persist, although it may be substantially lessened relative to physical terrorism). Instead, we argue that each terrorist event requires significant human involvement, e.g., to conceive, plan and execute it. This requirement for human effort implies that for the production rate of an organization to decrease, it must add additional members to produce them. And, the resultant increased rate occurs not because more hands make any individual event proceed more quickly, but because multiple events may be carried out in parallel. That is, the overall production rate of the organization is like the production rate of an entire factory; as the factory (organization) adds internal independent production lines (terrorist cells), the effective time between new events falls even though each production line operates at a constant rate.

It is important to recognize that H1 does not imply that the only way to increase the group-level production rate of attacks is through organizational growth. Indeed, many aspects of event production surely do benefit from technology or efficiency improvements [36]–[39]. Instead, H1 implies that such factors can only moderate, not eliminate, the fundamental constraint that size places on production. To the extent that these factors decrease the time between an organization's events, the literature on learning suggests that the overall impact will be modest [39]. In contrast, increases in labor, which allow many terrorist cells to operate in parallel, can lead to much larger improvements.

Finally, we note that this constraint should be strongest for small organizations, who likely have the worst access to efficiency-improving resources like specialized personnel, training facilities or factories and who may reap the largest benefit, e.g., media visibility, from striving to maximize their event production. Because most organizations begin small and grow over time, this should be most evidence early in the lifetime of an organization. (A spatial corollary of H1 is that if an “organization” is defined as those militants within some geographic locale, e.g., a province or district, then the frequency of events within that locale will be roughly inversely proportional to the number of militants there. That is, the 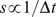 relationship should hold when both 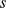 and 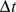 are defined by a geographic boundary. Organizational “growth” can then be understood as either immigration or recruitment of new militants).

### Events, Recruitment and Growth

What role do attacks play in changing organizational size? If an event gains the organization wider visibility among potential members or sympathizers, the organization may grow in size as a result of that event. (Decreases in size are likely driven by distinct social processes (see [40]), which we do not consider here).

H2 *Event-recruitment*: organizational growth (increased *s*) is partly driven by recruitment associated with the production of new events (increased *k*), i.e., events lead to recruitment which leads to organizational growth. Mathematically, *ds/dk>0.*

H2 does not imply that growth comes only from violence-related recruitment. So long as recruitment is partly based on the production of violent events, H2 implies a correlation between increases in size and increased event production.

### Frequency Acceleration

Together, H1 and H2 imply a positive feedback loop in which attacks lead to recruitment which leads to organizational growth and thus an increased group-level production of new attacks. So long as a portion of the growth is allocated to producing additional events, i.e., so long as the militant wing grows with the overall organization, H1 and H2 jointly imply H3.

H3 *Frequency-acceleration*: as an organization carries out more attacks (increased *k*), the time between subsequent attacks Δ*t* decreases. Mathematically, *d*Δ*t/dk<0.*

That is, H1 predicts 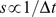 while H2 predicts 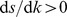. Eliminating the common factor of 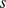 yields the prediction that 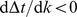, in which the continued production of violent events produces a decreasing delay between those events. (This dynamical relationship produces a similar pattern to that observed in “learning” or “progress curves,” in which continued production covaries with lowered production costs or time [36], [39], [41]. Although the pattern is similar, the mechanism is different).

### Impact of Size on Severity

Increased size may bring greater access to capital and skilled labor, e.g., experienced professionals, advanced arms, intelligence, etc., and thus more spectacular attacks.

H4 *Severity-increase*: the severity 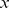 of a new attack increases with organizational size *s* and, via H2, the number of attacks *k*. Mathematically, *dx/ds>0* and *dx/dk>0*, respectively.

Combined with H2, H3 implies that attacks by experienced, larger groups should be consistently and significantly more deadly than those of less experienced or smaller groups.

H4 assumes a tangible benefit for maximizing the severity of attacks, e.g., to gain wider visibility for the organization's cause or to demonstrate power or resolve. Such incentives are not foregone conclusions: severe attacks may also attract harsh attention from state-level actors, leading to repression, police action or the destruction of physical or financial resources. They may also induce counter-productive effects on potential sympathizers, e.g., due to the shockingness of spectacular events. As a result, we consider the theoretical argument supporting the severity-increase hypothesis to be marginal.

## Results

### Model of terrorist organizations

**Figure 1:**
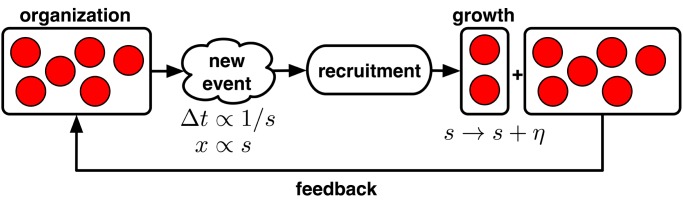
A model of terrorist organizations. A schematic illustrating the feedback loop relationship between size *s* and the frequency and severity of attacks: the delay between subsequent attacks Δ*t* is inversely related to an organization's size *s* while the severity of subsequent attacks *x* grows with *s*; new events lead to recruitment which leads to growth, which increases the size variable *s*.

To illustrate these interactions between an organization's size and the frequency and severity of attacks over its lifetime, we construct a simple model of a terrorist organization's development (see Figure 1 for a schematic).

Historically, terrorist organizations begin as a small collections of terrorism-inclined individuals [42]. Let this initial collection be composed of roughly 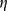 individuals, which denotes the typical or characteristic size of a terrorist cell. The particular value of 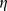 is not important, but may depend political ideology, socio-economic context [43], the attack's target, etc. The cell plans and conducts its first attack, which gains it some visibility, via either traditional media coverage or informal channels. Subsequent recruitment yields a number of additional members 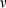 (H2), and now the organization is larger. Again, the particular value of 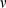 is not important, but likely depends on context-specific factors.

Each cell continues planning and carrying out new attacks, roughly once every 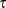 days (H1). Newly recruited members form new cells, of size 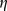 (H1) and new cells plan and carry out their own attacks in parallel. It is this parallelism that allows the larger organization to appear to be acting more quickly, even though the planning time 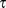 for any particular event remains fixed. An attack by any cell leads to overall organizational growth via recruitment (H2), which in turn increases the organization's overall production rate of attacks by adding new cells (H3). Finally, as the group grows, the increased manpower also increases its ability to carry out more severe events (H4), e.g., because more supporting roles allow better surveillance, access to better equipment, etc.

Coordinating the activities of these additional individuals, or the development of non-violent initiatives like a political wing or the provision of social services, will draw some members away from these militant activities. However, so long as recruitment continues to grow the number of militant cells, the positive feedback loop remains.

This simple model intentionally omits many factors, such as organizational structure, political motivation, geography, etc., that are likely to impact the behavior of any particular organization. We also intentionally omit any potential response by state-level actors and their consequences on the organization's evolution. This last decision is made in order to focus on the development of the organization, i.e., its early lifetime, where labor constraints are likely most profound, although such processes could naturally be added. Omitting these factors keep the model simple and allows us to make quantitative predictions of the generic relationship between organization size and the frequency and severity of its attacks via direct numerical simulation. To mimic the natural variation between particular events, for each new event being planned by a cell, we draw a delay 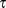 from a fixed distribution. (In general, our results hold so long as the distribution of 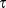 is well-behaved and stationary with respect to 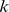.) Specification details and computer code for the simulation are given in Text S1.

Each simulated terrorist organization generates a unique sequence of events representing the collective behavior of its cells over time, and we extract the generic behavior by computing quantiles over variables of interest for many such simulated organizations. Here, we are interested in how the delay between subsequent attacks 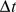 varies with cumulative number of events 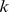 (H3), and how the size of the organization, measured by the number of cells 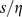 varies with calendar time 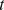 from the first event (H2). H4 predicts that event severity correlates with organization size and thus no additional information is gained by explicitly simulating event severities.

**Figure 2:**
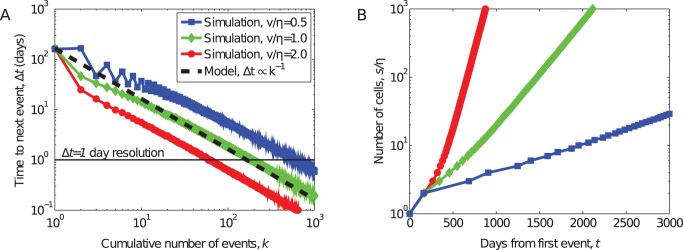
Simulated development of a terrorist organization. (A) Median event delay Δ*t* vs. cumulative number of events *k*, for 10,000 simulated terrorist organizations and three choices of the number of cells *v/η* added per event. Dashed line shows the function Δ*t∝k^−1^*, from Eq. (1). (B) Median size (number of terrorist “cells” *s/v*) vs. calendar time from the first event, showing exponential growth with rate set by *v/η*.

Figure 2 shows the results for 10,000 simulated organizations, for three choices of the ratio 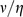, which represents the growth rate of the organization's militant wing. When 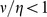 regime, organizational growth is slow because multiple events are required to establish a new terrorist cell; but, when 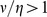, organizational growth is fast because each event produces at least one new cell.

The generic behavior of our model is clear: (i) organizational size grows exponentially with time, at rate 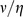, and (ii) the feedback between size and production rate induces a strong correlation between experience, size and the frequency of events. Finally, the model produces a universal functional relationship between delay 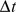 and cumulative production 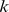 of the form 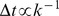, and this relationship is independent of the growth rate 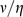.

This latter point is worth reiterating: so long as each new event leads to some marginal increase in the overall production rate (H2), a positive feedback loop between size and event production will exist. This feedback will be linear 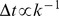 if the growth rate 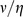 does not vary with experience 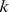. If the militant wing is a decreasing fraction of the overall organization (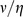 decreases over time), the feedback will be sub-linear and 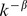 with 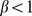, while if it increases with time, the feedback will be super-linear and 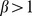. These properties imply that if a growing organization does provoke responses from state-level actors, these responses will not break the feedback loop unless they succeed in both limiting the growth and reducing the size of the organization, a point to which we will return later.

These quantitative predictions can be tested with empirical data by examining 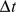 as a function of 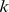 across many organizations. If 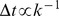 holds in the data, we have strong evidence for precisely the size-mediated feedback loop described here.

### Empirical data

Organizational size data were drawn from the Big Allied And Dangerous (BAAD) data set [8], which offers the currently best available size estimates for terrorist organizations worldwide. Other sources of size data lack the breadth or temporal resolution for accurate analysis. For instance, the START program and the MIPT database previously held a small number of estimates of uncertain accuracy, generated by Detica, Inc., a British defense contractor, and [44] compiled a database of information on 649 terrorist groups that included only estimates of the maximum size over a group's entire lifetime. The BAAD data were generated by a survey of domain experts at the Monterey Institute of International Studies (MIIS) who estimated the rough order of magnitude (1–100, 100–1000, 1000–10,000 and 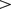
10,000 personnel) of the maximum size achieved by each of 381 groups, between 1998 and 2005, identified in the [45] event database. Of these, 161 organizations conducted at least one deadly attack, and 80 conducted at least two in that period.

To ensure good compatibility with this organization list, event data were drawn from the MIPT Terrorism Knowledge Base [45], which contained 35,668 terrorism events, of which 13,274 resulted in at least one fatality, as of 29 January 2008. (Other sources of event data include the Global Terrorism Database [46], the Worldwide Incident Tracking System [47] and the ITERATE data [48]. We note that neither these nor the MIPT database provide complete and consistent worldwide coverage.) For the period 1968–1997, the MIPT database includes mainly international events involving actors from at least two countries, while for 1998–2008 it includes both domestic and international events from much of the world. (The MIPT data were originally drawn from the RAND Terrorism Chronology 1968–1997, the RAND-MIPT Terrorism Incident database (1998–Present), the Terrorism Indictment database (University of Arkansas & University of Oklahoma), and DFI International's research on terrorist organizations. In 2008, however, the U.S. Department of Homeland Security discontinued its funding for the maintenance of the database in favor of the University of Maryland's START center's Global Terrorism Database [46].) Each event is defined as an attack on a single target in a single location (city) on a single day. For example, the Al Qaeda attacks in the United States on 11 September 2001 appear as three events in the database, one for each of the New York City, Washington D.C. and Shanksville, Pennsylvania locations. Each record includes the date, target, city (if applicable), country, type of weapon used, terrorist group(s) responsible (if known), number of deaths (if known), number of injuries (if known), a brief description of the attack and the source of the information.

The organizations identified in the MIPT database are a superset of those contained in the BAAD data set, and we will use these additional data analyses that do not require size estimates. For each organization, we extracted the full sequence of its attributed or claimed events. This yields 10,335 events worldwide from 1968–2008 associated with 910 identifiable organizations. For each of the 1,204 events worldwide with unknown severity, we assign a severity of 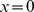 to preserve timing information. Further, because of the day-level temporal resolution of events in the database, multiple events on the same day by the same group have ambiguous “delay” (inverse frequency). We eliminate this ambiguity by aggregating such events into a single “event day” with severity equal to the sum of the component severities. This slightly reduces the number of events, mainly for the most active organizations late in their life history. As a consequence, the minimum resolvable delay in the database for two events by the same organization is 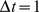 day.

### Regression models

Before analyzing the evolution of attacks by individual organizations we conduct static or cross-sectional regression analysis at the level of individual organizations. We examine the relationship between group size and attack patterns, in particular the delay between attacks, the experience of a group in terms of number of events, and the severity of attacks.

To recap, we expect larger groups to generate a larger number of attacks, have shorter delays between attacks (H1), and generate more severe attacks even accounting for other attack patterns (H4). We can evaluate H1 by comparing maximum group size 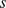 from BAAD and the minimum delay between attacks 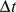 in MIPT. We can assess H4 by comparing size and the maximum severity 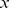 of attacks. Finally, H2 implies that larger groups should have higher maximum experience 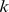 or cumulative number of events. (H3, postulating a declining delay with subsequent attack, cannot be evaluated with static data; we return to this point later).

Although group size should predict attack patterns, individual measures such as maximum severity will be at least in part a function of the total number of attacks. That is, for any distribution of severities, an increased production rate (sampling intensity) will naturally inflate the maximum severity over a fixed time period, even if the distribution is stationary. Thus, in order to examine the partial relationship between size and the related attack variables–or their independent predictive value on size once we take into account the other attack pattern characteristics–it is more convenient to consider to what extent we can account for size as function of the attack measures.

We use an ordered logit regression model of size since the BAAD data give order-of-magnitude estimates of maximum size. As the BAAD data pertain to the time period 1998–2005, we restrict our attack pattern measures to attacks during this same time period. Since the distributions of minimum delay, maximum experience, and maximum severity are all highly skewed we take the natural logarithm, adding 1 to severity to prevent taking the log of 0 in the case of non-fatal events. We report the empirical estimates in Table 1.

**Table 1:** Ordered logit regression of group size, by fatal attack patterns.

Variable	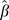	SE( 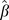 )
Delay: ln min(Δ*t*)	−0.351	0.119
Experience: ln max(*k*)	0.707	0.193
Severity: ln max(*χ*)	0.150	0.159
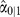	−0.163	0.840
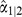	2.652	0.895
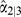	5.039	1.056

N = 80, LR *χ^2^* = 41.42, df  = 3, 58.75% correctly classified.

The results display a significant negative relationship between fatal attack delay and group size, consistent with our claim that larger groups will have shorter delays between attacks (H1). We also find a positive relationship between group size and experience, consistent with our claim that larger groups generate a higher number of attacks (H2). Finally, the maximum severity of the attacks is not significantly related to group size, once we have controlled for delay and experience variables. This contradicts the hypothesis that larger groups are systematically more likely to generate severe attacks (H4). Overall, the model places 58.75% of all the groups in the correct bins for group size. Only 5% of the observations are badly mis-classified, with predictions off by more than one order of magnitude. By contrast, a null model predicting all groups to have the modal size category (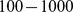) only classified 43.75% of the observations correctly. (We considered a number of alternative specifications. Severity remains an insignificant predictor of group size when we consider combinations of delay and experience for both deadly and non-deadly attacks. Using a linear regression model rather than ordered logit does not change our substantive conclusions).

Since the BAAD data cover only about half of the identifiable organizations in the MIPT database over a restricted time span (1998–2005), we conduct a supplementary analysis with the full MIPT dataset, where we consider how a group's total experience can be accounted for by differences in minimum delay and maximum attack severity. (We limit the analysis to MIPT organization that generated at least two events (frequency) and one deadly event (severity); only 167 organizations satisfy these criteria.) Table 2 report the results for a linear regression with logged values for all the terms for fatal (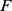) and all attacks (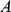, including non-fatal attacks) experience respectively. The results clearly show that the minimum delay is a significant predictor of group experience, and they mildly support the claim about severity, as the positive coefficient for severity is significantly different from 0. However, comparing the change in the R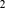 for estimating the model with and without the severity and delay terms respectively indicates that dropping the severity variable leads to a relatively small decline, while the impact of omitting the delay variables is substantial. Hence, variation in delay between attacks accounts for much more of the variation in experience than does severity.

**Table 2:** Linear regression of experience, by attack delay and severity.

	Fatal attacks (*F*)		All attacks (*A*)	
Variable	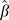	SE( 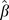 )	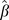	SE( 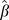 )
Delay*_F_*: ln min(Δ*t*)	−0.119	0.042	−0.110	0.040
Delay*_A_*: ln min(Δ*t*)	−0.778	0.110	−0.795	0.105
Delay*_F_*× Delay*_A_*	0.074	0.017	0.073	0.016
Severity: ln max(*χ*)	0.190	0.059	0.150	0.056
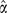	3.115	0.236	3.336	0.225
	N = 167, R^2^ = 0.545		N = 167, R^2^ = 0.565	
	R^2^ ( 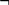 severity) = 0.515		R^2^ ( 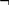 severity) = 0.546	
	R^2^ ( 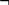 delay) = 0.222		R^2^ ( 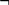 delay) = 0.182	

These static analyses provide substantial preliminary evidence in support of H1 and H2 and little evidence to support H4. We now go beyond static analyses and test our predictions for all organizations in the MIPT database using a novel dynamical analysis tool called a “development curve”.

### Developmental dynamics

A development curve is a statistical tool that measures the evolution of organization behavioral variables along a common quantitative timeline [22]. It is similar in structure and use to the “experience”, “learning” and “progress curves” sometimes used in management science [36], [39] to quantify the relationship between per-item production cost (or time) and “experience” (cumulative item production). Because we study behavioral variables rather than the costs of production, and to explicitly avoid implying learning-based mechanisms, we choose a distinct term. The analysis of these developmental curves facilitates direct comparisons of the behaviors of different groups at similar points in their life histories, which is useful for testing our hypotheses.

We instrument a common timeline using organizational experience 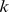, defined as the cumulative number of events produced by or associated with a particular organization, and we compare the delay 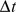 between the 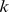
th and 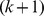
th events, or the severity 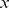 of the 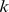
th attack, across all organizations in our sample. For each of the 910 organizations, we extract from the MIPT event data an ordered sequence of coordinates 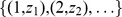, which represent the group's behavioral trajectory on the variable 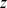 over its lifetime. The visualization of such trajectory is typically made using double-logarithmic axes, as illustrated in our simulation results in Figure 2. Although the curve construction itself ignores details such as the date of an organization's first attack, its location, ideology, etc., these variables can be used for subsequent analysis, e.g., comparing the trajectories across covariates.

Constructing a development curve for an individual organization (see Text S1) can facilitate the investigation of specific behavioral dynamics of individual groups over their lifetimes. However, the specific factors associated with particular organizations may obscure the generic tendency embodied by our hypothesis. To investigate these, we examine the average trajectory across many organizations by tabulating the conditional distribution 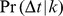 of delays, for a specified level of experience 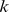. Thus, an organization that has carried out 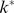 events contributes to each of the 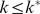 conditional distributions. This approach provides a strong test of the frequency-acceleration (H3) and attack-severity hypotheses (H4) predictions.

#### Frequency of attacks over time

**Figure 3:**
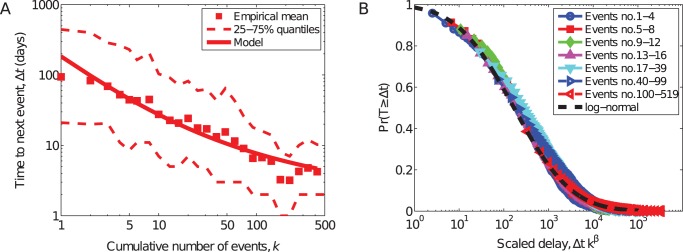
Timing of events. (A) Mean delay <*log *Δ*t*> between attacks, with 1st and 3rd quartiles, vs. group experience *k*. Solid line shows the expected mean delay, from the statistical model described in the text. (B) A “data collapse” showing the alignment of the re-scaled conditional delay distributions 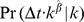 with the estimated underlying log-normal distribution, as predicted by the model.

Figure 3A shows the composite frequency curve for all organizations in our study. To reduce the overprinting effects of showing the trajectories for so many organizations, we bin the values of 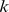 on a logarithmic scale and plot the mean and 1st and 3rd quartiles of the data within each bin. Remarkably, the observed empirical pattern agrees very closely with our simulation model's predictions (Figure 2).

The progressive decrease of the delay distributions indicates a generic tendency toward faster production with increased experience for all types of organizations, in strong agreement with the frequency-acceleration hypothesis (H3). But, the relationship between delay and experience is not deterministic: not every event occurs more quickly than the last but the statistical tendency toward shorter delays is clear.

A terrorist organization thus typically begins in the low-frequency domain (large 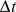) and moves in fits and starts toward the high-frequency domain (small 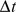). This trend is not subtle: the median delay after the 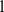
st event is 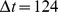 days, while by the 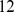
th event, it has dropped to 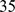 days and by the 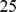
th, the next event typically comes only 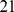 days later. This transition to fast production does take considerable calendar time: for groups that achieve 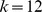 events, the median total calendar time between the first and twelfth event is 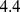 years. Similar results hold for the timing between deadly attacks.

None of the sampled organizations progressively slowed their attack rate over time, moving from high-frequency to low-frequency. A few unusual groups, such as Al-Qaeda in the Land of Two Rivers, begin and remain in the high-frequency domain. But, Al-Qaeda in the Land of Two Rivers is an interesting case because it is well-known to have operated under a different name prior to 2004 [49]; thus, their initial high-frequency behavior can be interpreted as support for the labor-constraint hypothesis (H1) because their initial larger size–a hold over from their previous identity–allowed them to “begin” life (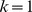) at a relatively high initial production rate of attacks.

#### Statistical model for the frequency of attacks

Quantifying the dynamical relationship between delays and experience allows us to go beyond our static analyses. To do this, we statistically model the conditional distribution 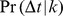 from which delays are drawn and how this distribution varies with experience.

For these data, a truncated log-normal distribution, with the following mathematical form
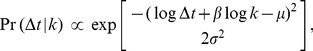
(1)provides an excellent fit to the empirical delay data for all organizations. Here, 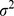 is the variance in delays at a given 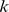, 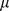 is related to the characteristic delay between attacks and 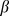 controls the rate at which that delay decreases with increased experience 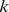. That is, 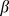 governs the strength of the feedback loop between organizational experience and the production of new events. To include the effect of the minimum timing resolution 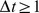 present in the empirical data, we force 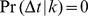 for 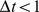 day.

This mathematical structure implies that the typical delay between attacks generically decreases according to a power-law function with increasing experience.
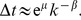
(2)


(Details of this derivation are given in Text S1.) Thus, if 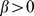, we will observe a transition toward increasingly fast event production, indicating support for H3. In contrast, if 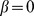, production rates do not vary with organizational experience, while if 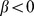, production rates will decrease (larger 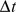) with increasing experience. In the 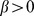 regime predicted by H3, the acceleration effect is dampened as the mean delay asymptotes to the minimum timing resolution at 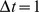; this produces slight upward curvature for large values of 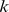 (see Text S1).

The particular value of 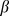 has a strong effect on the material dynamics of the feedback loop between increasing experience and increasing production. If 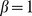, then the feedback loop is linear, as in our simulation model, and increases in organizational experience lead to proportional increases in event production. Linearity implies that the marginal growth associated with an additional event is relatively constant over the organization's lifetime and a roughly constant fraction of new recruits are allocated to increase overall tempo of militant activities.

In contrast, *β≠1* implies a non-linear feedback process. Notably, non-linear feedback processes are not common models of social processes (but see the literature on arms-races, particularly [17] and [50]). Traditional models often focus on proportional effects in which increases in one variable cause proportional changes in other variables. In non-linear feedback processes, small increases in one variable can produce dramatic and continuing swings in other variables, leading to highly unpredictable dynamics [51].

When 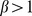, the feedback is super-linear, and one or both of these factors must increase with 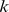. That is, either per-event growth in militant activities increases over time or an increasing fraction of growth is allocated to militant activities. When 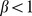, the feedback is sub-linear and the marginal recruitment benefits of new events decrease over time or they are constant but recruits are increasingly allocated toward non-militant activities.

Fitting this model directly to the empirical data on all events, we find that the maximum likelihood estimate is 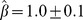 (std. err.), indicating linear feedback. (This approach to estimating the parameter gives weight to the events early in organization's lifetime that is proportional to the number of such events in our data set; in contrast, a simple regression approach on the mean delays would bias the estimate by giving significant weight to the rare but long-lived groups.) Using a Monte Carlo simulation against a null model with fixed 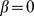 (no acceleration over time) and with 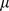, 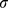 estimated using maximum likelihood given the fixed 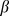 value, we find that the value of 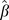 is highly statistically significant (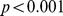). (Fitting to deadly attacks alone yields a highly statistically significant 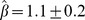, slightly in the super-linear regime, but this value is statistically indistinguishable from 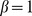).

A linear feedback implies that the marginal growth from event-driven recruitment does not vary much with organizational size or experience. Furthermore, it implies that organizational learning in terrorist groups [25], [38], in which the production rate increases due to improved efficiency of a fixed number of individuals, plays a lesser role in explaining the overall acceleration of event production than do the effects of increasing organizational size, because learning would mimic the effect of super-linear feedback by allowing a constant number of militants to behave identically to an increasing number.

A strong test of the statistical model's plausibility is its prediction that each of the 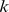 conditional delay distributions 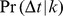 is a scaled version of the underlying log-normal distribution LN(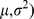. To test this prediction, we re-scale the empirical distributions by the predicted factor, i.e., we multiply each delay variable 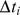 by 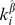, and then plot them against the estimated underlying log-normal distribution. A close alignment of these re-scaled conditional distributions, also called a “data collapse” [52], is strong evidence for the hypothesized data model over a wide range of alternatives. Furthermore, for an alternative model to produce such a data collapse requires that it follows the log-normal form closely enough to be effectively equivalent. Figure 3B shows the results of this test, illustrating an excellent data collapse, with each of the re-scaled log-normal conditional distributions closely aligning with the underlying log-normal form.

These results also hold when we consider the development curves for groups with a common political ideology (see Text S1). [53] divides the political motivations for terrorism into four conventional categories: nationalist-separatist, reactionary, religious and revolutionary. We coded according to Miller's criteria the 131 most prolific groups in our sample (all with 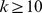 deadly events), which accounts for 85% of events, and fitted Eq. (1) to the data within each ideological category. Organizations with multiple political motivations were placed in multiple categories, which would only lessen any differences between estimated parameters for different categories. Within each of these categories, we observe the same acceleration pattern, with the strongest acceleration (largest 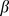) appearing for religious groups (Table 3).

**Table 3:** Frequency curve parameters for organizations with similar political motivations.

political motivation	groups	events	*μ*	*σ*	*β*	significance
nationalist- separatist	55	2959	5.1(5)	2.2(1)	0.9(2)	*p*<0.001
reactionary	5	143	3.2(6)	1.8(2)	0.1(3)	*p*<0.001
religious	17	999	5(1)	2.4(5)	1.7(5)	*p*<0.001
revolutionary	53	2527	5.7(4)	2.3(2)	1.1(2)	*p*<0.001
all secular	883	6232	5.2(2)	2.25(9)	0.9(1)	*p*<0.001
all groups	910	7231	5.1(2)	2.32(9)	1.0(1)	*p*<0.001

Note: statistical significance estimated via Monte Carlo simulation of a two-tail test against a null model with *β = 0* (no frequency acceleration), using the sum-of-squared errors (SSE). Values in parentheses indicate bootstrap standard uncertainty in the last digit.

#### Severity of attacks over time

**Figure 4:**
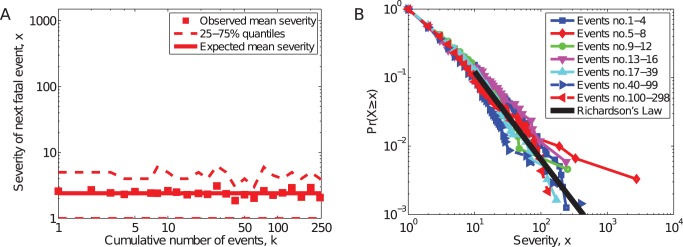
Severity of events. (A) Mean severity <log *x*> of deadly attacks, with 1st and 3rd quartile, vs. group experience *k*. Solid line (with slope zero) shows the expected delay, from a simple regression model. (B) Conditional severity distributions Pr(*x/k*), showing a data collapse onto a heavy-tailed distribution, with the maximum likelihood power-law model for all severities (Richardson's Law).

In contrast to the delay development curve, we find no statistically significant relationship between the severity of attacks and increased experience (Pearson's 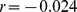, t-test, 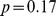), indicating no support for the severity-increase hypothesis (H4). Across all organizations in our sample, the average severity of the first deadly event is 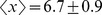, which is only slightly larger than the average severity of deadly events by highly experienced groups (those with 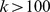) 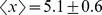. Figure 4A shows the composite severity curve for all organizations in our study.

As with the frequency curves, we find that the conditional severity distributions 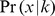 roughly collapse onto a single, underlying form (Figure 4B), which is similar to the power law observed for all deadly terrorist attacks worldwide from 1968–2008 [20], [31]. That is, Richardson's Law for terrorism appears to hold for both inexperienced and highly experienced groups. Combined with our static analysis of organizational size, this pattern implies a highly counter-intuitive fact: the severity of attacks by larger, more experienced organizations, is not significantly greater than the severity of attacks by small, inexperienced organizations. That is, the common assumption that only experienced groups are capable of such mass destruction [54] is incorrect: inexperienced organizations are just as likely to produce extremely severe events as highly experienced organizations.

However, although more experienced organizations are not systematically more lethal at the individual-event level, the observed frequency-acceleration pattern implies that more experienced groups are significantly more lethal overall. This pattern was observed by [8] in their analysis of the BAAD organizations. Our results thus clarify their results, showing that the observed correlation between greater lethality (total deaths attributed to an organization) and greater organizational size appears because larger, more experienced organizations produce events more quickly than smaller, less experienced organizations. It is the cumulative effect of the many small events that generates an increased lethality, not a systematic increase in the lethality of individual events.

Repeating this analyses on our ideology-coded set of organizations, we find no systematic dependence of severity of attacks on organizational experience within any of the ideological categories (see Text S1). That is, none of the model coefficients are significant, and the average severity of events within each category vary only a little. In short, we find that political ideology has no systematic impact on the severity of events or the trajectory that event severities take over the lifespan of an organization.

## Discussion

Although details and circumstances vary widely across terrorist organizations, the generic nature of our results suggests general conclusions. In particular, we find strong evidence for a positive feedback loop among organizational size (number of personnel), experience (cumulative number of events) and the frequency at which that organization launches new events. Small and inexperienced organizations tend to produce events slowly, while larger and more experienced organizations tend to produce events sometimes hundreds of times more frequently.

Within this feedback loop, new attacks lead to organizational growth and the corresponding increase in size leads to faster production of new events because a larger size means more terrorist cells are operating in parallel, not because events themselves are planned more quickly. The result of this feedback loop is a generic “developmental” trajectory: as an organization ages, it tends to produce violent events more and more quickly.

The typical form of this relationship can be mathematically modeled by a power-law function, in which the delay 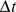 between consecutive events decreases roughly like 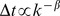 where 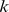 counts the cumulative number of events and 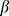 describes the strength and direction of the feedback loop. The implication of the power-law pattern is that large organizations are very much like “scaled up” versions of small organizations, and in particular that size and experience are coupled in a positive feedback loop.

Across all organizations in our sample, we estimate 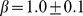, indicating a linear feedback loop, which implies that an organization's overall size is strongly correlated with the size of its militant wing. This pattern is strongest for small or inexperienced organizations, e.g., those with 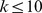 events, which covers 87% of the 910 organizations in our sample. In contrast, highly experienced organizations seem to saturate their event production rates at the daily or weekly level, which may be indicative of a tendency of large organizations to engage in multiple types of activities, e.g., the provision of social services, criminal activities, etc., continuing to grow their militant wings.

The mathematical precision of this relationship is striking, as is the ability of our computer simulation to reproduce it. Except for Richardson's Law for the frequency and severity of wars, few statistical relationships in the study of political violence exhibit such regularity.

The power-law relation between organizational experience and production rate is both conceptually and mathematically similar to the relationship between cost and cumulative production observed in manufacturing [36] or organizational learning [37], [39], where decreases in per-item production costs or time can be described by a power law in the cumulative number of items produced. That a similar patterns appears in the production of terrorist events is surprising, and it may not be superficial to describe terrorist organizations as a special type of manufacturing firm whose principal product is political violence and whose overall production of violence is fundamentally constrained by its size.

The implication is that terrorism is inherently non-amenable to mass production, i.e., it is not a scalable enterprise, perhaps because each event must be humanly conceived and planned around a particular target, tactic or environment, and there is a limit to how much this process can be automated. One implication of this conclusion for cyber-terrorism is that even there, despite the great potential for automating attacks, these too will likely not be scalable without advances in general artificial intelligence.

In the language of economics, we say that terrorism capital and labor are not freely substitutable with respect to producing new events. If the day-to-day work of event production does not require specialized skills, then the growth potential of an organization be extremely large because it may draw on the largest possible pool of potential recruits. This point suggests that conflict-level event production rates should ultimately be responsive to policy and counter-terrorism efforts that target the size and mobility of the pool of potential recruits. That is, successful “hearts and minds” strategies [55] are likely to lead directly to lower incident rates by both restricting the growth and reducing the size of terrorist organizations. They may not, however, eliminate the possibility of spectacular attacks as these do not depend on organizational size.

Recently, following our original work on progress curves in terrorism [22], Johnson et al. [25] analyzed the timing of events in the Iraq and Afghanistan conflicts, finding similar power-law like acceleration curves in the delay between events. They argue that this pattern is caused by a kind of “red queen” effect–a concept borrowed from arms races in evolutionary biology [56]–in which two sides of the conflict race through some abstract space, and the timing between events is given by how far “ahead” the insurgent side is in the race. In practice, however, this explanation is difficult to validate because the connection is not specified as to how real-world events and structures drive the dynamics of the abstract race. In contrast, our explanation of the phenomena is both tangible, general and testable: we argue that the size of the insurgency or the terrorist group sets the tempo of the conflict. The more people there are fighting, the more frequently we will observe events. This explanation makes direct and testable predictions about the relationship of organizational size and frequency of events, which we show are upheld by empirical data on organizational sizes. (As a technical note, in the language of physics, the “size” of an organization or insurgency is an extensive variable of the conflict system, much like area and number of particles are for physical systems [57]; this fact makes additional testable predictions of our theory.) The implication for the Iraq and Afghanistan conflicts is that the number of insurgents active in the various provinces is the primary determinant of the frequency of events observed there.

Although the acceleration is remarkably strong, the vast majority of organizations do not achieve high levels of experience (only 23% of groups are associated with 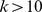 events) or fast production rates. The progressive loss of organizations could be due to high rates of organizational death, e.g., from counter-terrorism activities or internal conflicts [44], [58], shifts away from violence, or a right-censoring effect on young and still active organizations. Significantly, the particular mode of organizational demise seems not to have a strong impact on the production time of events, suggesting that the transition from development (growth) to death may happen very quickly, so that the experience curve does not bend upward but rather simply halts. Further exploration of the death of organizations [44], [58], and how it impacts the production of violence, is an interesting avenue for future work.

Regardless of the reason, we do not expect the feedback loop to continue as 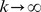. If an organization succeeds in becoming large enough to produce new events each day, it may function more like a stable or mature social institution, with fundamentally different constraints and incentives on the production of violence. Large size and stability may also pose special risks, e.g., leading to larger or longer conflicts. On the other hand, non-violent activities, e.g., engagement with political processes, may also become more attractive with increased size. Exploring these possibilities is an interesting avenue for future work.

Unlike the production of events, we find no evidence of any relationship with the severity of attacks (H4). Rather, Richardson's Law–a power-law distribution in the frequency of severe events–characterizes the severity of events at all levels of organizational size or experience, and independent of the organization's political ideology.

This fact clarifies ongoing efforts to identify the underlying social, political or physical mechanism that generates Richardson's Law in terrorism. Several existing explanations assume or predict a severity-size relationship, e.g., the aggregation-disintegration model of Johnson et al. [23] and [35], but these seem increasingly unlikely given our results here, because they assume the maximum severity of an event is proportional to the organization's size 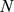; thus, if 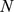 is small, the severity of events 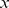 will also be small. That is, in their existing form, these models predict a severity-size relationship that does not appear in the data. Of course, these models may be adapted to produce the observed size-independence pattern, but doing so requires additional assumptions and additional validation that may not be warranted.

In contrast, two plausible explanations are not ruled out: (i) the explanation proposed in [20], which posits a coevolutionary competition between states and terrorists in which event planning time and severity are strongly related, and (ii) the explanation proposed in [24], in which population densities are broad-scaled and terrorists preferentially target high-density locations. Both of these explanations do not assume any relation between the severity of an attack and the size of an organization.

Together, our results suggest that the total lethality of larger and more mature groups observed by Asal and Rethemeyer [8] is probably best explained as a natural consequence of their much more frequent activities, rather than as a systematic increase in the deadliness of individual events. Policies that limit the growth of an organization's militant wing should lower the long-term probability of a severe event by that organization. Such growth-limiting policies could be described as “starving the beast” of the labor necessary to produce rare but highly severe events.

The most productive targets of such policies will be large, established organizations with long histories of producing terrorist attacks. By virtue of their size, these organizations are likely to be well-known players in their particular conflicts and thus easy targets for specific policies. Because small organizations are equally likely to produce severe events, policies aimed specifically at large, well-known organizations may not limit the overall risk of severe events from all sources. For small and potentially unknown organizations, the most effective policies may be those aimed at preventing their formation in the first place, i.e., policies that curtail the acquisition of the means for and resort to violence. Lacking this, once such a terrorist cell carries out its first attack and begins its developmental trajectory, the best action by a government may be an “overwhelming response” to encourage through various means the dissolution of the nascent organization and the truncation of its growth trajectory. This policy is not without risk to the state, however, as certain countermeasures may serve the terrorist's goals [59], [60].

In closing, we point out that the acceleration in the frequency of terrorist events is independent of many commonly studied factors associated with terrorism, including geographic location, time period, international vs. domestic targets, ideological motivations (religious, national-separatist, reactionary, etc.), and political context. Our results thus demonstrate that some aspects of terrorism are not nearly as contingent or unpredictable as is often assumed and the actions of terrorists may be constrained by processes unrelated to strategic tradeoffs among costs, benefits and preferences. Identifying and understanding these processes offers a complementary approach to the traditional rational-actor framework, and a new way to understand what regularities exist, why they exist, what they imply for long-term social and political stability, e.g., large-scale violent conflicts like civil and interstate wars.

## Supporting Information

Text S1 Supplementary Text. 1. Additional analysis of organizational sizes and their event frequency and severity. 2. Frequency and severity development curves for four highly prolific organizations. 3. Specification and code for the simulation model. 4. Mathematical details of generic pattern in event frequencies versus experience. 5. Robustness checks of the frequency-acceleration pattern. 6. Analysis of developmental trajectories of organizations by political ideology.
(PDF)
