# Supplementary material for: The Developmental Dynamics of Terrorist Organizations
Source: PLoS One. 2012 Nov 21;7(11):e48633. doi: 10.1371/journal.pone.0048633 (PMC3504060; doi:10.1371/journal.pone.0048633)
Supplement: Text S1 — Supplementary Text. 1. Additional analysis of organizational sizes and their event frequency and severity. 2. Frequency and severity development curves for four highly prolific organizations. 3. Specification and code for the simulation model. 4. Mathematical details of generic pattern in event frequencies versus experience. 5. Robustness checks of the frequency-acceleration pattern. 6. Analysis of developmental trajectories of organizations by political ideology. (PDF) [file pone.0048633.s001.pdf]

The developmental dynamics of terrorist organizations
Supporting Information

Aaron Clauset [1,2,3,*] and Kristian Skrede Gleditsch [4,5]

[1] Department of Computer Science and
[2] BioFrontiers Institute, University of Colorado, Boulder CO, USA
[3] Santa Fe Institute, Santa Fe NM, USA
[4] Department of Government, University of Essex, Wivenhoe Park, Colchester, UK
[4] Centre for the Study of Civil War, Oslo, Norway

[*]To whom correspondence should be addressed. E-mail: aaron.clauset@colorado.edu.

Section A *Supplemental Analysis of Size, Frequency and Severity*: Additional analysis of the organizational size data, with respect to the frequency and severity of their events.

Section B *Development Curves for Four Prolific Organizations*: Individual frequency and severity development curves for the four most prolific organizations in the MIPT dataset.

Section C *Terrorist Organization Computer Simulation*: Specification and simulation code for the computer simulation described in the main text.

Section D *Statistical Model for the Frequency of Attacks* : Mathematical details of the statistical model for the generic pattern in event frequencies versus organizational experience.

Section E *Domestic vs. Transnational Events* : Robustness check of the frequency acceleration pattern by considering organizations whose first event was prior to 1998 (mainly international terrorist organizations) versus after (mainly domestic terrorist organizations).

Section F *Political Ideology & Frequency and Severity Curves* : Variation in the developmental trajectories of organizations by political ideology, showing different frequency acceleration rates and no differences in event severity evolution.

A Supplemental Analysis of Size, Frequency and Severity

The growth hypothesis predicts that a groups maximum size will be inversely related to the minimum delay between its attacks over the 1998–2005 period. To complement the analysis in the main text, here we show the graphical plots and conduct additional analysis.

An analysis of variance indicates that the average minimum delays differ significantly between size categories (n-way ANOVA, $F = 9.98$, $p < 0.000013$). Further, we find that larger organizational size is a significant predictor of increased attack frequency ($r = -0.49$, t-test, $p < 10^{-5}$). Fig. S1a shows the distributions within the size categories. Although the distributions do overlap somewhat, the downward trend is clear.

In contrast, size, like experience, is not a significant predictor of median attack severity (n-way ANOVA $F = 0.59$, $p = 0.62$). Fig. S1b shows the distributions within the period. (We choose medians because they are robust to the large fluctuations caused by small samples drawn from heavy-tailed distributions.) Although there is some variability between size categories, the lack of a trend is clear.

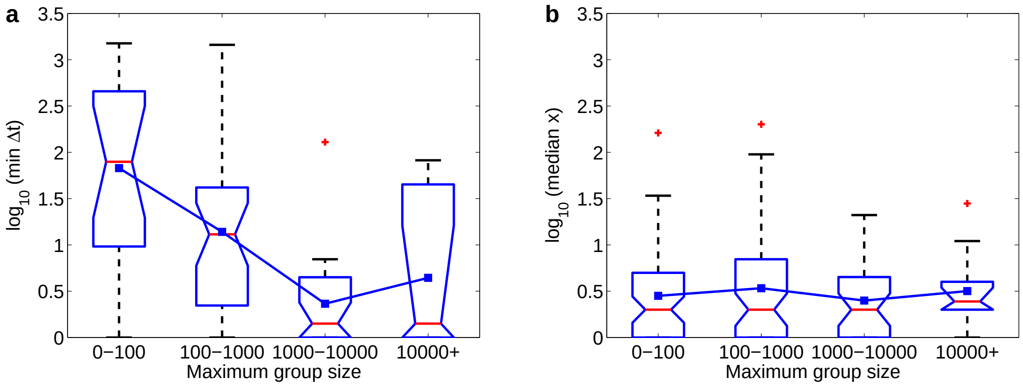

Figure S1: Box-plots of the distributions of a groups (a), minimum delay $\log(\min \Delta t)$ and (b), median attack severity $\log(\text{median} x)$ for attacks within 1998–2005, within each of four size categories. For convenience, we connect the means of each category, which are significantly different in the case of delays (n-way ANOVA, $F = 9.98$, $p < 0.000013$), but indistinguishable in the case of severities (n-way ANOVA, $F = 0.59$, $p = 0.62$).

B Development Curves for Four Prolific Organizations

As an example of development curve analysis, Figure S2 shows the frequency and severity development curves for the four organizations with the greatest number of attributed event-days in our dataset, including both deadly and non-deadly events: the Revolutionary Armed Forces of Colombia (FARC; 520 events), the Taliban (349 events), Basque Fatherland and Freedom (ETA; 311 events), and Hamas (308 events). Non-deadly events ($x = 0$) increment the counter k for the severity curve but do not appear on the severity curve figures; hence, ETA, which carried out 261 (84%) non-deadly events, has relatively few points in its severity curve.

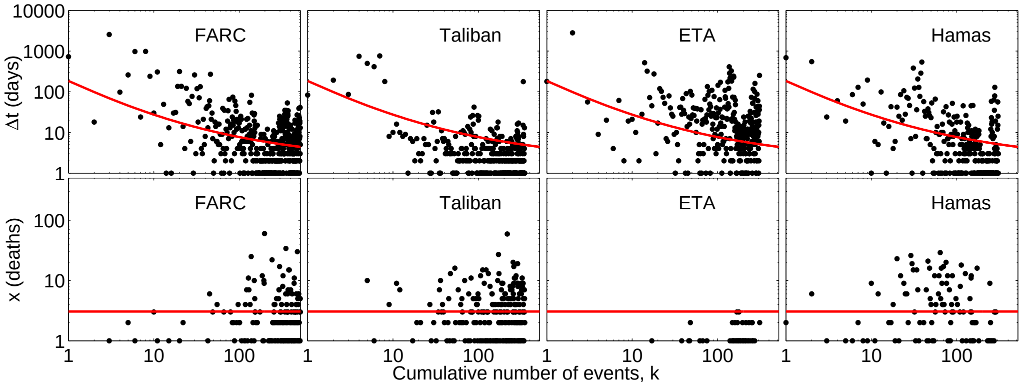

Figure S2: Frequency (delay Δt) and severity (deaths x) development curves for the Revolutionary Armed Forces of Colombia (FARC), Taliban, Basque Fatherland and Freedom (ETA), and Hamas, with generic trajectories estimated for all groups. Similar results hold for less experienced groups.

For these organizations, the median delay between the $k = 1$ and $k = 2$ events is $\Delta t = 433$ days. In contrast, the median delay between the most recent pair of events by these groups is only $\Delta t = 4$ days, a 100-fold increase in frequency. In each case, the frequency curve begins in the upper-left corner of the figure, representing very long delays between subsequent events, and as k increases, the curve moves consistently, albeit stochastically, toward the bottom-right corner, representing a convergence on very short delays between events.

This progression from slow to fast event production appears to happen quickly: each of these groups achieves delays of $\Delta t \leq 10$ days by their $k = 12$th event. However, the median calendar time required to achieve this high rate of production is 8.5 years; thus, although these first dozen events account for a small fraction of the lifetime production of these organizations (less than 4% each), they account for a large fraction of the organizations' overall lifetimes. Put more bluntly, these first few events play a critical role in shaping the long-term trajectory of an organization's production curve and they illustrate a dramatic acceleration in the production of events as the organizations mature. This important developmental effect is obscured by high production rates later in life.

In contrast, the pattern for the severity development curve could not be more different: we observe no clear trend, either up or down, between event severity x and experience k for these organizations, and the median first and last severities are $x = 0$ and $x = 1$ deaths, respectively. If anything, the only visual pattern we can discern is a possible increase in the variance of x as k increases. This preliminary analysis thus already indicates weak support for the severity-increase hypothesis (H4) but strong support for the frequency-acceleration hypothesis (H3). In combination with our static analysis above, this provides additional evidence supporting labor constraints and event-driven recruitment (H1 and H2).

C Terrorist Organization Computer Simulation

The toy model described in the main text can be formalized and simulated explicitly. Below is computer code that implements the simulation in Matlab. In words, the simulation works as follows.

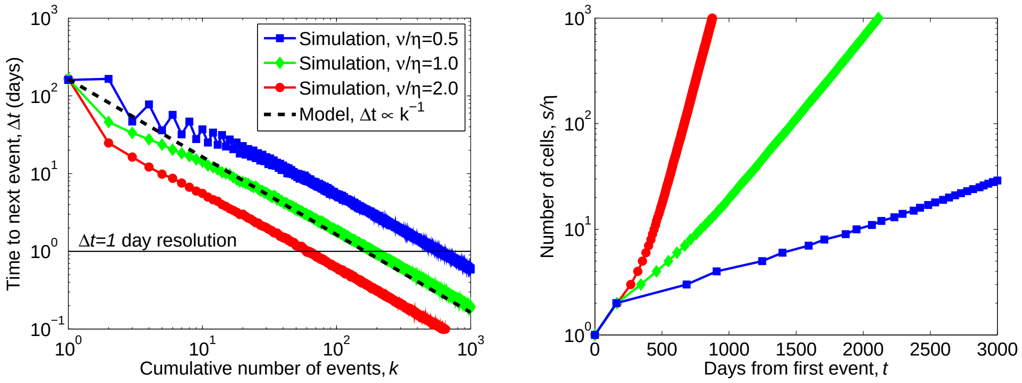

Figure S3: (a) Median event delay Δt vs. cumulative number of events k, for 10,000 simulated terrorist organizations and three choices of the number of cells ν/η added per event. Dashed line shows the function $\Delta t \propto k^{-1}$, from $\Pr(\Delta t\,|\,k) \propto \exp\left[\frac{-(\log\Delta t+\beta\log k-\mu)^2}{2\sigma^2}\right]$. (b) Median size (number of terrorist "cells" s/ν) vs. calendar time from the first event, showing exponential growth with rate set by ν/η.

Let η be a constant that denotes the number of individuals that make up a terrorist "cell" within the organization, and let ν be the number of individuals the organization as a whole gains via recruitment after each event. Thus, η/ν events are required to produce a single new cell; the particular values of η and ν serve only to change the scale of the dynamics, not their fundamental character. Each cell is assigned a "clock" that measures the number of days remaining before that cell generates an event. We denote this delay τ and draw it from a log-normal distribution with parameters μ and σ, i.e., $\Pr(\tau) \sim \mathrm{LN}(\mu,\sigma)$. This is the only stochastic element of the simulation. When a cell generates an event, it then draws a new delay from the same distribution.

As described in the main text, each organization begins as a single cell, which has generated a single event at $t = 0$. Thus, initially $s_1 = \eta$. We then choose a delay τ for its next event. The simulation will generate a specified number of events, specified by the parameter `nok`. For the kth event, the simulation then checks which cell has the smallest remaining delay and advances all cells' clocks by that much. It then generates the kth event, records its time as an ordered pair (k, t_k), and draws a new clock value for the generating cell. Additionally, it increments the organization's size by ν individuals, i.e., $s_k = s_{k-1} + \nu$, and adds $\lfloor s_k/\eta \rfloor$ new cells, each with a clock drawn from $\Pr(\tau)$.

A number of variations of this model generate equivalent results. For instance, the distribution $\Pr(\tau)$ can generate very small delays, e.g., less than 1 day, which may be considered unrealistic. Imposing a minimum value on the $\Pr(\tau)$ does not change the fundamental feedback between size and event production and thus leaves the k^{-1} trend unchanged. And, the ratio η/ν only re-scales the underlying k^{-1} behavior, as seen in Figure S3. Finally, changing the parameters of $\Pr(\tau)$ has no impact on the fundamental behavior: the μ parameter sets the delay between the first and second events, which appears as the expected y-intercept on the resulting development curve, and varying σ simply changes the scatter around the underlying trend. In fact, the particular functional form of $\Pr(\tau)$ we have chosen is not important, and other choices lead to similar results; here, we choose the log-normal distribution due to its similarity to the empirical data (Fig. S4).

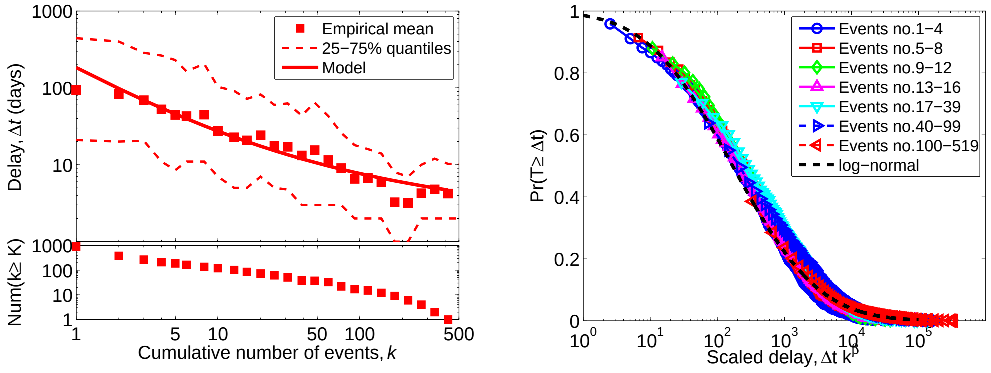

Figure S4: (a, upper) Mean delay $\langle \log \Delta t \rangle$ between attacks, with 1st and 3rd quartiles, vs. group experience k. Solid line shows the expected mean delay, from the statistical model described in the text. (a, lower) Number of organizations with at least k events. (b) A "data collapse" showing the alignment of the re-scaled conditional delay distributions $\Pr(\Delta t \cdot k^{\hat{\beta}} \mid k)$ with the estimated underlying log-normal distribution, as predicted by the model.

```
% --- Terrorist organization simulation
% --- by Aaron Clauset

% --- set up simulation parameters
[mu  sigma] = deal(5.1,2.32); % parameters for Pr(tau) = LN(mu,sigma)
[eta nu]    = deal(5,5);      % size of cell,  marginal growth after an attack
nok         = 1000;           % number of events to generate

% --- set up simulation data structures
s   = zeros(nok+1,1); % organization size over time
c   = s;              % number of cells over time
[s(1) c(1)] = deal(eta,1);
fk  = zeros(nok+1,2);
fk(:,1) = (1:size(fk,1))';    % assign ids to events
gr      = zeros(nok+1,2);     % holds event clocks for each cell
gr(:,1) = (1:size(gr,1))';    % assign ids to cells

% --- initialize simulation: create the first cell
t   = 0;                      % global clock
k   = 1;                      % number of attacks to date (first attack at t=0)
tau = exp(sigma*randn(1)+mu); % choose delay from Pr(tau)
gr(1,:) = [1 tau];            % make first cell

% --- generate exactly nok events
while k<size(fk,1)

    % -- advance time to next attack
    [dt i] = min(gr(1:c(k),2)); % find cell with next attack
    t      = t + dt;            % advance all clocks by that much time
    gr(1:c(k),2) = gr(1:c(k),2) - dt;

    % -- generate the kth event
    k        = k + 1;           % increment attack number
    fk(k,2)  = t;               % record time of this event
```

```
    tau = exp(sigma*randn(1)+mu);
    gr(i,2) = tau;              % choose new delay for this cell

    % -- recruitment / growth
    s(k) = s(k-1) + nu;         % grow total personnel
    c(k) = floor(s(k)/eta);     % count no. cells
    dc = c(k) - c(k-1);         % calculate cell growth
    if dc>0                     % create the new cells and choose their delays
        tau = exp(sigma*randn(dc,1)+mu);
        gr(c(k-1)+1:c(k),2) = tau;
    end;

end;

% --- done generating events; extract results
[dt k] = deal(diff(fk(:,2)),(1:size(fk,1)-1));

% --- plot resulting development curve
figure(1); clf;
loglog(k,dt,'r-','LineWidth',2); hold on;
loglog([1 nok],exp(mu).*([1 nok]).^(-1),'k--','LineWidth',3); hold off;
xlabel('Cumulative number of events, \it{k}','FontSize',16);
ylabel('Time to next event, \Delta\it{t} \rm{(days)}','FontSize',16);
set(gca,'FontSize',16,'YTick',10.^(-6:4));
h1=legend(strcat('Simulation, \nu/\eta=',num2str(nu/eta,'%3.1f')), ...
     'Model, \Deltat\propto k^-^1',1); set(h1,'FontSize',16);
```

D Statistical Model for the Frequency of Attacks

The probabilistic model for event delays used in the main text, given by Eq. (1), has the precise form of

$$\Pr(\Delta t \,|\, k) = \left(\frac{\sqrt{2/\pi}}{\sigma \left(1 - \mathrm{Erf}\left[\frac{\beta \log k - \mu}{\sigma\sqrt{2}}\right]\right)} \right) \exp\left[\frac{-(\log \Delta t + \beta \log k - \mu)^2}{2\sigma^2}\right] \tag{1}$$

where the leading term is the normalization constant and $\mathrm{Erf}(\cdot)$ is the error function. In words, this model asserts that the logarithm of the delay Δt is a random variable distributed according to a Normal distribution $\mathcal{N}(\nu, \omega)$ (or equivalently, the delay is log-normally distributed) with a lower cutoff at $\Delta t = 1$ day (to reflect the timing resolution of the event data), constant variance ω and a distributional mean ν that decreases systematically with increasing experience k. In Eq. (1), the parameter μ denotes the characteristic delay between attacks, and in particular the delay between the first and second attacks, while σ^2 denotes the variance in the expected delay.

The equation given in the main text for the expected delay as a function of experience—the central tendency of the conditional distribution of delay as a function of experience—can be derived in the usual way. Doing so yields

$$\mathrm{E}[\log \Delta t] = \mu - \beta \log k + \left(\frac{\exp\left[\frac{-(\beta \log k - \mu)^2}{2\sigma^2}\right] \sqrt{2/\pi}}{\sigma^{-1} \left(1 - \mathrm{Erf}\left[\frac{\beta \log k - \mu}{\sigma\sqrt{2}}\right]\right)} \right) \quad , \tag{2}$$

which has a simple leading form and a complicated trailing term. For small values of k, the expected delay is dominated by the leading two terms, i.e., the trailing term is small in relative magnitude, and thus

the trend is well-approximated by a power-law function $\Delta t \approx \mathrm{e}^{\mu}\mathrm{k}^{-\beta}$, where e^{μ} represents the initial rate of attack of a group. At larger values of k, the expected delay is dominated by the trailing term, which makes the expected delay to approach $\Delta t = 1$ more slowly than a power law.

When fitting this model to the empirical data, we estimate its parameters using standard numerical procedures to maximize the likelihood of the data (in this case, the Nelder-Mead 1965 method). Standard error estimates for the uncertainty in the parameters are then estimated using a bootstrap procedure on the organizations in the sample.

The striking "data collapse" shown in Figure 3b illustrates that the conditional probability distributions do indeed align closely with the estimated log-normal model for delays. Why delays should be log-normally distributed remains a mystery.

Finally, we point out that very few groups (e.g., Hamas, Fatah, LTTE, FARC, etc.) manage to become highly experienced ($k \gtrsim 100$). This means that the fit of the model for large-k is primarily controlled by the delays at much smaller values of k, where the vast majority of the data lay. This fact explains the slight misfit of the model to the delays for highly experienced groups. However, it also highlights the fact that the behavior of inexperienced groups early in their lifetime is highly predictive of the behavior of mature organizations.

E Domestic vs. Transnational Events

From 1968–1997, the MIPT event database was maintained by RAND as part of its project on transnational terrorism. As a result, almost no domestic terrorist attacks are included before 1998, after which the scope of the database was significantly expanded (in part due to the Oklahoma City bombing in 1995) to include purely domestic events worldwide. Although organizations and events are not coded as being transnational or domestic, the inconsistency in database scope provides an opportunity to test whether the frequency dynamics of domestic terrorism organizations differs from those of transnational organizations.

By dividing events into those generated by organizations whose first event occurred 1968–1997 and those generated by organizations whose first event occurred in 1998–2008, and then repeating the frequency-curve analysis from the main text, we may test whether the frequency-acceleration phenomena appears only in one time period or the other. Further, because events in the 1998-2008 period are mainly domestic events, while those in the 1968–1997 period are only transnational events, the two time periods serve as proxies for transnational-only and domestic-only terrorism. This division does not control for non-stationary effects.

Figure S5 shows that the development curve phenomenon is robust to this division, indicating that the frequency-acceleration appears to hold for both transnational and domestic terrorism. One difference between these time periods does emerge: the rate of acceleration for the 1968–1997 data (transnational only) is $\hat{\beta}_{t_1 \leq 1997} = 1.0 \pm 0.2$ (stderr), statistically indistinguishable from the analysis of all organizations in the main text, while the estimated acceleration for the 1998–2008 data (mainly domestic) is slightly faster, with $\hat{\beta}_{t_1 > 1997} = 1.3 \pm 0.2$. The origin of this difference may be related to the increasing frequency of religiously-motivated terrorism in the 1990s and beyond (2; 3), who collectively exhibit a lower value of $\hat{\beta}$ than other types of terrorism. An interesting alternative explanation, however, is that some non-stationary process is having a consistent upward pressure on β over time, for all organizations. One

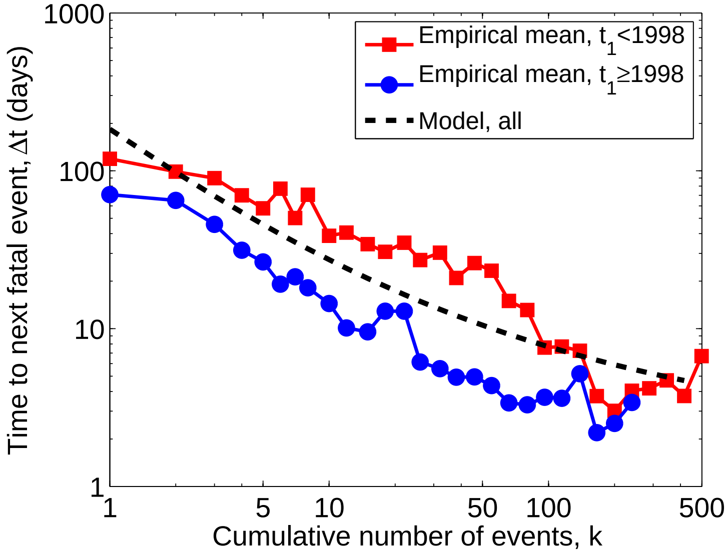

Figure S5: The attack frequency development curves, plotted as the average delay versus experience, for groups whose first attack was in 1968–1997 versus those whose first attack was in 1998–2008, along with the model estimated for all events from the main text.

candidate process is the development and spread of modern communications and digital technology, which may enable more widespread or effective recruiting efforts and thus faster organizational growth.

F Political Ideology & Frequency and Severity Curves

Our results for the developmental dynamics of event frequency and severity are good descriptions of the generic behavior of terrorist organizations. However, we have so far omitted any role for organizational covariates, many of which are believed to have important impacts on organizational behavior and decisions (see (4; 5; 6), among others). We investigate this question by studying the impact, if any, political or ideological motivation may have on the frequency curve's structure; we leave the investigation of other covariates for future work.

Miller (7) divides the political motivations for terrorism or group ideologies into four conventional categories: nationalist-separatist, reactionary, religious and revolutionary. We coded according to Miller's criteria the 131 groups in our sample with $k \geq 10$ deadly events, who together account for 85% of events (the majority of our data), and fitted Eq. (1) to the data within each ideological category. Organizations with multiple political motivations were placed in multiple categories, which would only lessen any differences between estimated parameters for different categories. Fig. S6a shows the corresponding central tendencies, as described by Eq. (2). Table 3 summarizes the estimated parameters for each ideological category and groups overall.

We again test the statistical significance of the acceleration effect within each ideological model using a two-tail test against a null model with fixed $\beta = 0$ (no acceleration over time). In all cases,

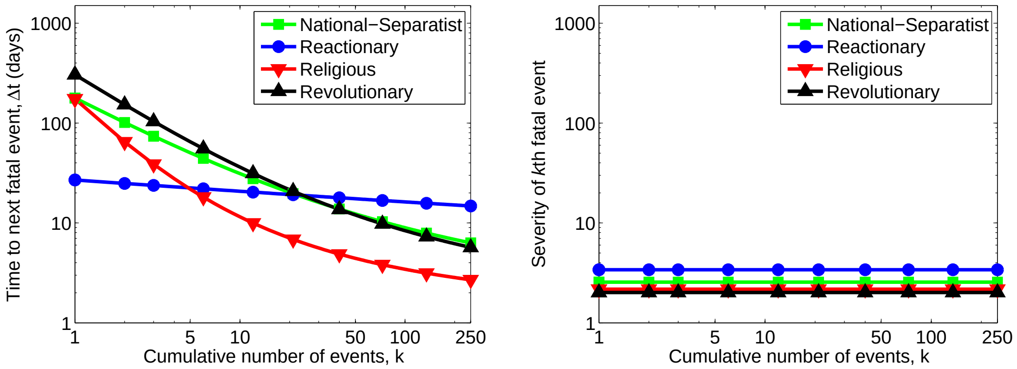

Figure S6: (a) Estimated frequency curves for four ideological categories, showing that religious groups develop extremely quickly relative to other types. (b) Estimated severity curves for the same categories, showing the same pattern of independence as Fig. 4a.

the estimated β parameter is highly statistically significant (at the $p < 0.001$ level), indicating that the acceleration within each category is real.

Among the four ideological categories, we observe wide variation in the estimated values of β and thus in the strength of the feedback loop governing the frequency of attacks. Religious groups have the largest value at $\hat{\beta} = 1.7 \pm 0.5$, placing them firmly in the super-linear feedback regime and implying very strong acceleration in the frequency of attacks over time. In contrast reactionary organizations have the smallest at $\hat{\beta} = 0.1 \pm 0.3$, placing them strongly in the sub-linear regime. Revolutionary and nationalist-separatist categories are statistically indistinguishable from the linear-feedback regime of $\beta = 1$.

The typical religious group, i.e., one accelerating along the generic production trajectory identified above, with $k = 10$ deadly attacks, attacks as frequently as the typical revolutionary group with $k = 51$ deadly attacks or the typical nationalist-separatist group with $k = 129$ attacks. When viewed in terms of calendar time, this difference is even more striking: it takes the typical religious terrorist organization only 400 days (1.1 years) to generate its first 10 attacks and at this point its production rate is approximately one attack every 5 days. In contrast, the typical revolutionary organization takes 1666 days (4.6 years), more than four times as long, and a typical nationalist-separatist organization takes 2103 days (5.8 years), to achieve an equal production rate. Combining this insight with the results of our static analysis on the role of size, the explosive acceleration by religious groups implies that they grow in size extremely quickly, which is the ultimate cause of their dramatic production rates.

But religious organizations are not universally more dangerous. Comparing the $\hat{\mu}$ parameters, which governs the characteristic delay between subsequent attacks, we observe a more complicated story: reactionary groups initially attack the fastest, with the fitted model estimating typically $\Delta t = 47$ days between their first and second attacks, while all other groups take substantially longer ($\Delta t > 100$ days). This difference in initial production rates is quickly eliminated by the explosive acceleration of religious groups as well as the more measured development of revolutionary and nationalist-separatist organizations, whose typical event production rates overtake that of reactionary groups after between 5 and 25 events.

Table S1: Severity curve parameters for organizations with similar political motivations. Note: statistical significance calculated using a t-test on Pearson's correlation coefficient.

political motivation	groups	events	$\langle x \rangle$	r	significance
nationalist-separatist	51	1003	6.1	0.0071	$p = 0.75$
reactionary	5	77	7.1	0.1194	$p = 0.27$
religious	17	753	5.2	-0.0062	$p = 0.49$
revolutionary	41	725	5.1	-0.0109	$p = 0.38$
all groups	381	3143	7.3	-0.0240	$p = 0.17$

Much previous work on religious terrorism has argued, largely on theoretical grounds, that such organizations are fundamentally more dangerous than secular groups (7; 8; 9; 10) because they have fewer social restrictions on their activities and are thus more free to produce and target violence than secular organizations, whose victims may be potential sympathizers. Our results provide indirect support for this argument, in the sense that religious organizations exhibit explosive acceleration in the production of violence while secular organizations exhibit more moderate acceleration.

However, arguments that religious organizations are universally more dangerous may have over-simplified organizational behavior by ignoring how organizations may change their behavior over time and how they vary relative to other organizational types. We find that very early in their life histories, religious groups are in fact less dangerous than reactionary groups, and only slightly more dangerous than national-separatist or revolutionary groups. It is only over the long term that the explosive acceleration experienced by religiously-motivated organizations allows them to cumulatively produce so many more events than other types of organizations. That is, only if a religious organization succeeds in reaching a more mature state does it pose a greater overall risk than groups with secular motivations. And, it is important to note that historically speaking, most organizations do not live so long (11): fully 55% of organizations in the MIPT database are associated with only a single event.

Turning briefly to the question of how event severity varies with organizational ideology, we repeat the same severity-curve analysis on the deadly events produced by the 131 highly prolific organizations. Figure S6b shows the resulting ideology-specific severity curves and Table 4 summarizes the estimated model parameters, where the model now is a simple linear regression of severity x against experience k. As above, we find no systematic dependence of severity of attacks on organizational experience within any of the ideological categories. That is, none of the model coefficients are significant, and the average severity of events within each category vary only a little. Thus, we find that political ideology has no systematic impact on the severity of events or the trajectory that event severities take over the lifespan of an organization.

References

[1] Nelder J, Mead R (1965) A simplex method for function minimization. Computer Journal 7: 308–313.

[2] Rapoport DC (2004) Modern terror: The four waves. In: Cronin AK, Ludes JM, editors, Attacking Terrorism: Elements of a Grand Strategy, Washington D.C.: Georgetown University Press. pp.

46–73.

[3] Rosenfeld JE (2011) Terrorism, Identity and Legitimacy: The Four Waves Theory and Political Violence. London: Routledge.

[4] Asal V, Rethemeyer RK (2008) The nature of the beast: Organizational structures and the lethality of terrorist attacks. Journal of Politics 70: 437–449.

[5] Pape RA (2003) The strategic logic of suicide terrorism. American Political Science Review 97: 343-361.

[6] Clauset A, Heger L, Young M, Gleditsch KS (2010) The strategic calculus of terrorism: Substitution and competition in the Israel-Palestine conflict. *Cooperation & Conflict* 45: 6–33.

[7] Miller GD (2007) Confronting Terrorisms: Group motivation and successful state policies. Terrorism and Political Violence 19: 331–350.

[8] Rapoport DC (1984) Fear and Trembling: Terrorism in three religious traditions. American Political Science Review 78: 658–677.

[9] Hoffman B (1995) "Holy Terror": The implications of terrorism motivated by a religious imperative. Studies in Conflict & Terrorism 18: 271–284.

[10] Juergensmeyer M (1997) Terror mandated by god. Terrorism and Political Violence 9: 16–23.

[11] Cronin AK (2009) How Terrorism Ends. Princeton NJ: Princeton University Press.
